# Influence of Stacking Sequence on Strength and Stability of Suspension System Control Arm CFRP Laminate Rods

**DOI:** 10.3390/ma14195849

**Published:** 2021-10-06

**Authors:** Paula Mierzejewska, Jacek Świniarski, Tomasz Kubiak

**Affiliations:** Department of Strength of Materials, Faculty of Mechanical Engineering, Lodz University of Technology, 90-924 Lodz, Poland; jacek.swiniarski@p.lodz.pl (J.Ś.); tomasz.kubiak@p.lodz.pl (T.K.)

**Keywords:** suspension system, wishbone, control arm, composite materials, CFRP, ply orientation, laminate layout

## Abstract

The paper deals with buckling and strength analysis of suspension system rods made of carbon fibre reinforced polymer (CFRP) laminate. The whole suspension system of urban solar vehicle, Eagle Two, designed by Lodz University of Technology students was considered. The calculations and analysis focused on suspension rods, where the traditional metal material was replaced with CFRP laminate. The influence of layer arrangement on rod strength, static, and dynamic buckling were analysed. The research was conducted using numerical simulations employing finite element method software. The static and dynamic load was considered. The obtained results show that the plies’ order in the laminate influences both the strength and stiffness of the considered rod. The best results considering both failure force and longitudinal elasticity modulus were obtained for the stacking sequences with axially oriented (0°) plies on the outside of the rod.

## 1. Introduction

A suspension system is one of the essential mechanical assemblies in vehicles. It consists of multiple elements linking load-bearing vehicle construction (frame or monocoque) to each wheel. It is responsible for carrying loads such as vehicle weight, forces due to propelling, braking, turning, and reactions to the road surface unevenness. Due to its construction, the suspension minimises vertical displacements of a car body and transverse tilts, providing good controllability and stability of vehicle motion, independent movement of each wheel, and constant contact between the wheels and road surface.

The intensive research on modern materials conducted by scientific centres around the world initiated studies not only on suspension optimisation regarding geometry but material as well. There emerged materials that can effectively compete with contemporarily used metal alloys—fibre reinforced composites. These materials can provide the same or even better mechanical properties (such as stiffness, strength, or wear resistance) compared with simultaneously decreasing the mass [1,2,3,4,5].

As far as composite suspension system components are concerned (control arms, in particular), few research articles are available. Anandakumar et al. l [6] considered the development of a short and continuous glass fiber reinforced composite control arm. The research showed its superior performance compared with steel and short fibre-reinforced composite component. In his article, Savage [7] explored sub-critical crack growth in suspension system elements of Formula 1 race car. In addition, Wang et al. l considered the substitution of metal alloyed suspension wishbone with fibre-reinforced composites. The study shows that the application of carbon and boron epoxy composites reduces weight at a level of 46%. There are also published research papers regarding numerical optimisation of composite control arms similarly leading to component weight reduction [2,5,8,9,10]. Considering automotive-related articles, only Ali et al. l [4] researched the influence of fibre orientation on the design of composite suspension system control arm.

Due to the increasingly growing popularity of fibre-reinforced composite materials in the automotive industry and yet a limited number of scientific articles, the authors considered the topic of a vehicle suspension system. The proposed research investigated the possibility of the application of composite materials in place of steel upper control arm of solar vehicle front suspension and studied the influence of laminate ply orientation on the mechanical behaviour of the proposed construction (including stress–strain state and buckling) under the application of different types of loading: static and dynamic (due to passing an assumed road bump). Considering the available literature, the article covered a thus far unexplored research topic regarding composite suspension system push-rods (constituting upper control arm) and their axial loading, including buckling analyses.

The research was performed employing numerical simulations—finite element method software.

At present, CFRC has become increasingly popular in the automotive industry, and consequently, its application is being investigated, and the results are published. Yet, the number of articles concerning the application of composite materials in vehicles’ suspension system components and their optimisation is still limited.

## 2. Object of Analysis

The subject of the research was a suspension system of the urban solar vehicle, Eagle Two, designed by a group of Lodz University of Technology students—Lodz Solar Team for the sake of international solar car races around the world, such as Bridgestone World Solar Challenge. Since its establishment in 2014, the team has participated in multiple races in Australia, South Africa, and Belgium, aiming to emerge the most energetically efficient urban solar vehicle with the highest comfort and utility. Therefore, mass reduction is of particular significance, which impacts energy consumption and driving comfort due to reduced unsprung mass oscillation.

**Figure 1:**
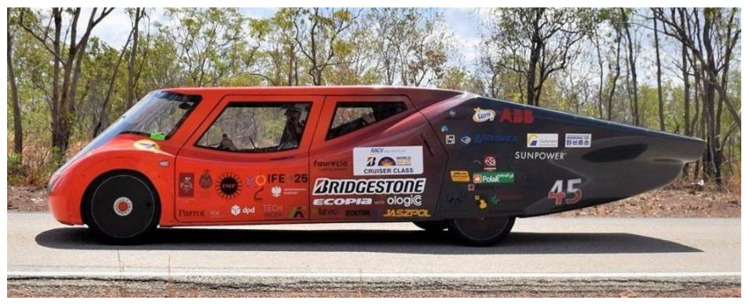
Eagle Two during BWSC 2017.

Eagle Two is a 5-passenger vehicle of total weight equal to 600 kg, powered by a 60 kWh lithium-ion battery and driven by 2 brushless direct-current motor (BLDC) electric motors with a total power of 10 kW. The electric energy is supplied to the battery by 5 m^2^ of photovoltaic panels of 24% efficiency, and the vehicle can also be charged using an electric charger. Eagle Two is characterised by a very low aerodynamic drag coefficient (c_x_ = 0.22), which allows the car to drive up to 1800 km on one battery charge. Figure 1 presents Eagle Two during the Bridgestone World Solar Challenge race in 2017.

The existing suspension system of Eagle Two consists of metal-alloyed elements only. Except for various advantages, metal alloys are also characterised by some features that constitute a problem. A change of construction by eliminating metal alloys and substituting them with composite materials would allow suspension system mass reduction, increasing the effectiveness, and comfort of driving.

The front axis of the vehicle is based on a double-wishbone suspension system with perpendicular control arms, whereas the upper control arm is substituted by two rods of different lengths. The front axis is not a propeller—both electric motors are placed in the rear wheels. Except for suspension system components, the front axis consists of a steering system and two independent braking system components.

**Figure 2:**
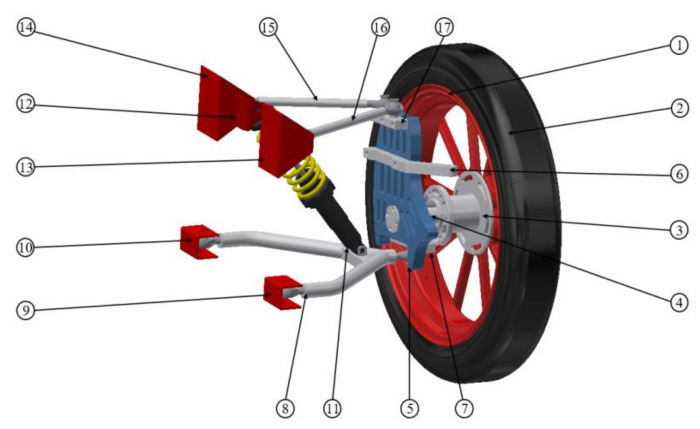
Schema of front suspension system (1—rim, 2—tire, 3—hub, 4—driveshaft, 5—steering knuckle, 6—steering linkage, 7—lower wishbone holder, 8—lower wishbone, 9, 10, 12–14—the connecting elements of suspension to the car chassis, 11—shock absorber with spring, 15,16—upper control arm, and 17—upper rod holder).

The front suspension system presented in Figure 2 consists of the wheel with the aluminium rim, aluminium hub placed on two bearings allowing smooth rotation on the driveshaft fixed to the steering knuckle. It is then mounted to the upper and lower control arms using spherical bearings. The upper control arm consists of two separate rods, which are the main object of investigations. Both wishbones are connected to the composite chassis using spherical bearings and metal mountings. The lower wishbone is connected to the chassis with spring–shock absorber assembly as well.

## 3. Numerical Models

Finite element analyses were performed in Hyperworks software (Altair Engineering Inc., Troy, MI, USA). Numerical models were prepared in Hypermesh (Altair Engineering Inc., Troy, MI, USA) preprocessor, whereas calculations were performed in Optistruct solver (Altair Engineering Inc., Troy, MI, USA).

The numerical suspension model was used to analyse suspension behaviour under static and dynamic load, while the model of the control-arm rods was used to perform analysis and research on the possibility of fibre reinforced polymer (FRP) laminate application as a material for this structure.

**Figure 3:**
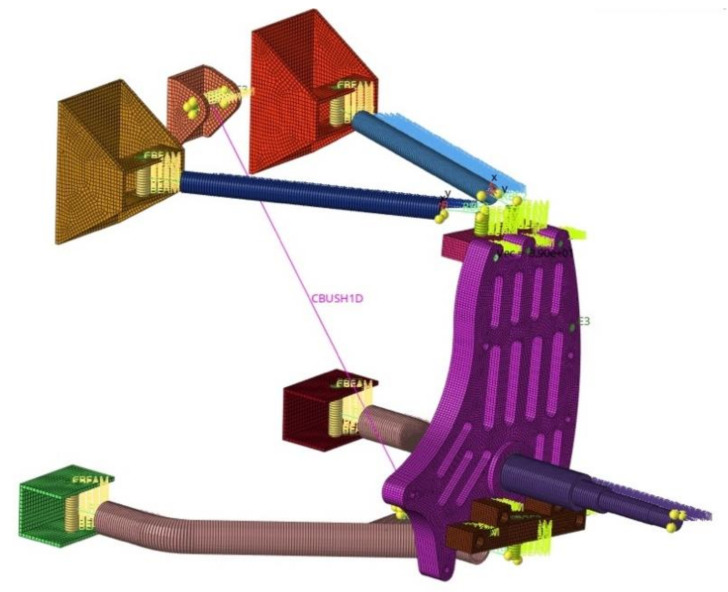
Mesh of the front suspension system.

The numerical model of the whole front suspension system is presented in Figure 3. The mesh was prepared for wishbones, steering knuckle, driveshaft, spring–shock absorber assembly, connectors, and mountings.

**Figure 4:**
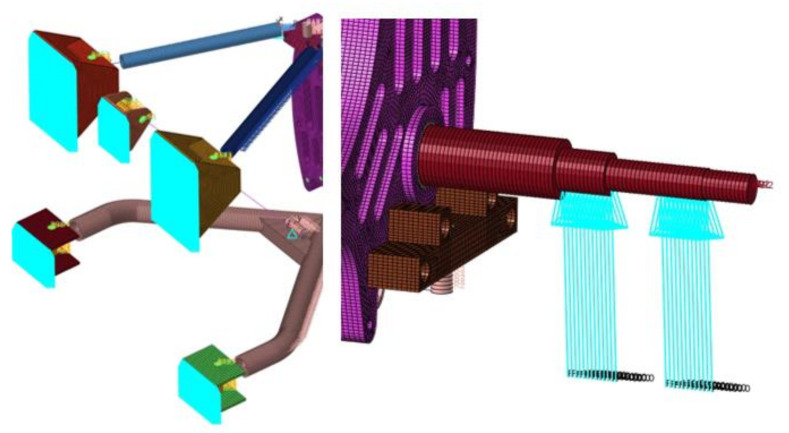
Suspension system numerical model—boundary and initial conditions.

The numerical model was prepared using both 1D 2-node beam elements and 3D 8-node solid elements, and the connectors (bolts and ball joints) were modelled using RBE2 and RB3 elements. These multi-point constraints define a rigid body with one independent node and arbitrary dependent nodes (RBE2) and motion at dependent node as the weighted average of independent nodes’ motions (RBE3). The spring–shock absorber assembly was prepared using a 1D spring-damper element (spring stiffness k = 57 N/mm, damping coefficient c = 2.7 Ns/mm). The suspension system was mounted to the chassis by constraining 6 degrees of freedom (DOF) at each of the mountings, whereas the loadings were placed on the nodes of the driveshaft corresponding to the placement of bearings (Figure 4).

The research was focused on the investigation of composite rods’ behaviour under axial loading. For that purpose, a carbon fibre reinforced composite design of the rods was proposed.

The original rods of the upper control arm are manufactured with S235 steel and are characterised by 16 mm external and 12 mm internal diameter. Their length is 196 mm for the first rod and 180 mm for the second rod. The mass of the rods is 0.135 kg and 0.124 kg, respectively.

**Figure 5:**
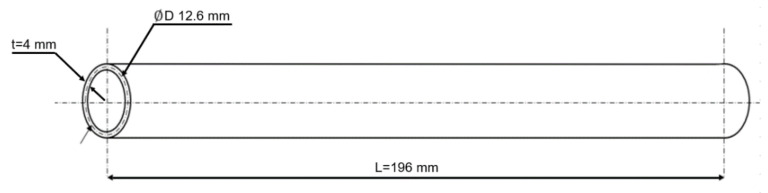
Schematic drawing and dimension of the rod under consideration.

The current construction of the rods is not threatened by the loss of stability due to buckling (in both elastic and non-elastic states) due to very low slenderness—39 for the longer (first) and 36.0 for the shorter (second) rod. Because of the considerable strength margin and based on the initial numerical calculation, it was decided to decrease the width of the rod walls for further research on the effect of laminate characteristics on load-bearing capability. We decided to reduce the external diameter of the rods, while the internal diameter and their lengths remained unchanged. Figure 5 presents the shape and dimension (D = 12.6 mm—mid-thickness dimension, t = 0.6 mm—wall thickness, and length of the rod L = 196 mm and 180 mm for longer and shorter rod) used in numerical models of the composite rods.

After redesigning, the slenderness of both composite rods is 44 and 40, respectively. The boundary slenderness values depend on the material properties, and therefore, for composite material, they will be different from those for steel. However, in case of such small values, it is probable that the buckling will not occur in the elastic region. The composite will be subjected to failure before a loss of stability because buckling occurs.

Considering the above, carbon fibre and epoxy resin composite in the form of a prepreg (Epoxycarbon UD395GPA from ANSYS composite materials library, ANSYS, Canonsburg, USA) was chosen. Such an epoxy-carbon composite is characterised by magnificent mechanical properties in the direction of fibres. Its tensile and compressive strength are a couple of times higher than steel, which makes this material suitable for a considered application. The material properties of the composite material are presented in Table 1.

The width of one laminate ply is equal to 0.1 mm. Based on initial analyses for further research, 6 layers in total—2 of direction 0°, 2 of direction 90° and 2 of direction 45°/−45°—were assumed. All of the considered laminate stacking sequences are presented in Table 2.

**Figure 6:**
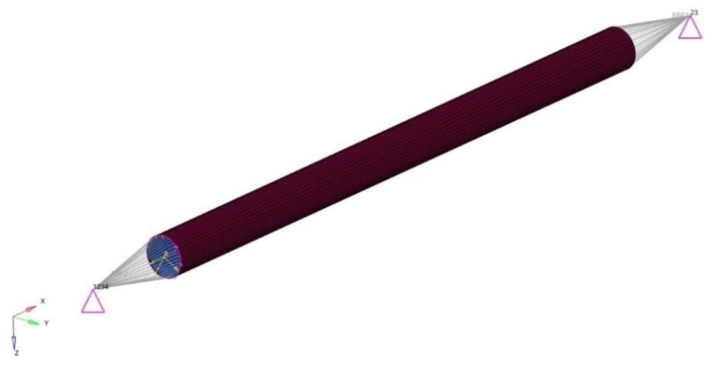
Composite rod numerical model.

For analysis, a numerical model of both rods was prepared. The rods were mounted accordingly to the suspension system—3 translation DOFs were taken on one side and 2 translation DOFs on the other side, except for x-direction (Figure 6).

The compressive load was applied to the end of the rod at which the longitudinal movement was allowed.

## 4. Results of Numerical Calculations

The numerical results obtained for whole front suspension systems and two rods of the upper control arm are presented below.

The analysis of the whole front suspension system was performed to check the entire system behaviour. Those results allow considering the proper loading applied to both suspension rods under investigations of layer arrangement influence on strength, static, and dynamic buckling behaviour.

### 4.1. Front Suspension System

Firstly, we performed a geometrically (large displacements) non-linear quasi-static analysis of the suspension system under the loading due to maximum vehicle mass 2500 N (250 kg) per front wheel. This analysis of the entire suspension system was used to check whether the relations between the force loading the suspension and the forces in the bars are linear.

**Figure 7:**
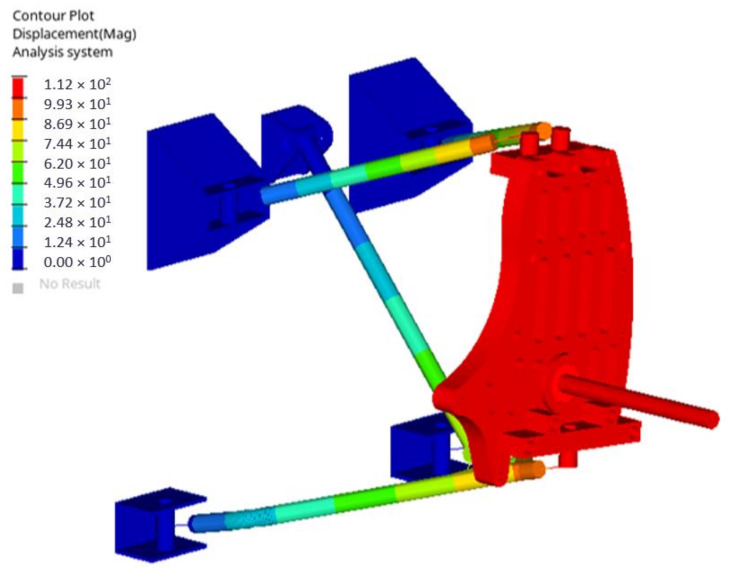
Suspension system displacement under loading of 2500 N.

The analysis results showed that the maximum displacement in the suspension system reached 111.7 mm and is related to the spring-damping element, which, under the loading, shortens and allows displacement of other suspension system components (Figure 7).

**Figure 8:**
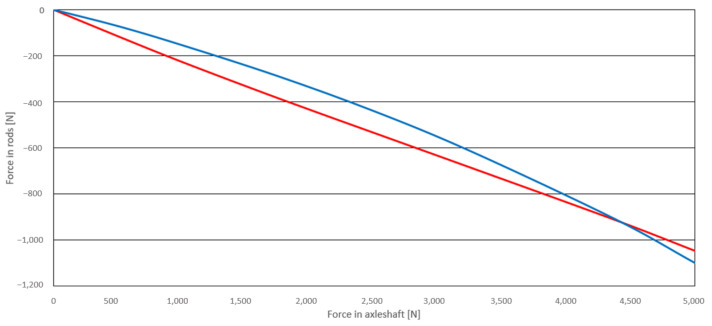
Compressive force increment in both rods as a function of wheel loading (blue—for rod 1, red—for rod 2).

According to FEM simulations, the rods of the upper control arm are subjected mainly to normal stresses. Shear stresses in the components are negligibly small. The normal stress for the first, longer rod equals –5 MPa, and for the second, shorter rod –6 MPa (negative signs indicate the compressive character of the loading). The forces responsible for causing normal stresses are for both rods 423 N and 537 N, respectively. Figure 8 presents the relationship between wheel loading and rods’ normal reaction forces. The analysis, therefore, showed that this relationship is not linear, especially for rod 2.

**Figure 9:**
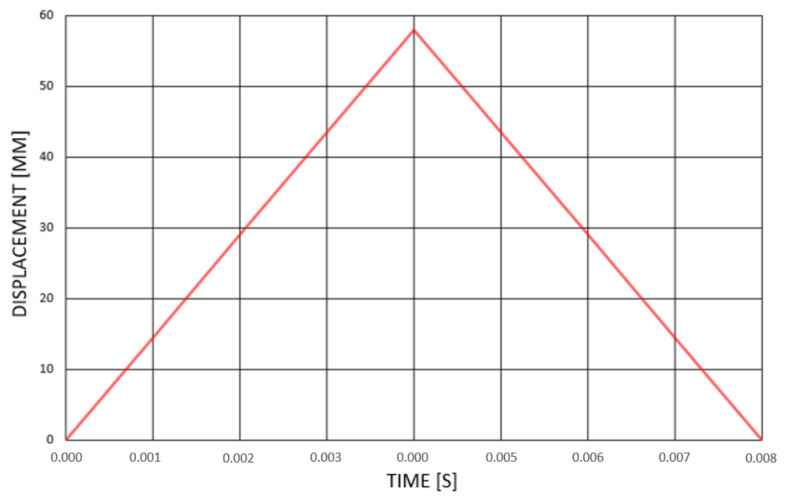
Displacement loading in time.

Reaction forces due to static loading are relatively low compared with the material yield strength; however, the suspension system is not only designed to withstand static loads. Its primary function is to carry dynamic loads. Therefore, a non-linear transient dynamic analysis was performed due to passing an exemplary road bump with the height of 58 mm (maximum capability of the vehicle suspension system) with the assumed speed equal to 45 km/h = 12.5 m/s. Hence, the total impulse time was equal to 0.008 s. The loading was applied as a displacement in time on the driveshaft. The dynamic impulse was assumed as a triangular shape with linear increasing and decreasing displacement—presented in Figure 9.

**Figure 10:**
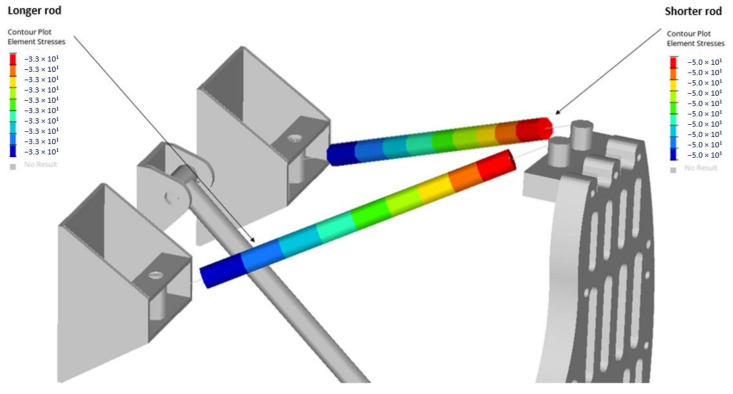
Maximum stresses in rods.

As a result, maximum compressive stress was obtained equal to 33 MPa for the longer rod and 50 MPa for the shorter one (Figure 10).

**Figure 11:**
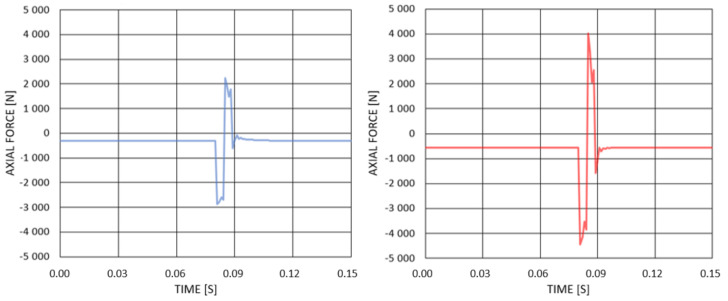
Axial force in rod 1—blue, axial force in rod 2—red.

Figure 11 presents the normal reaction forces in both rods as a function of time. This behaviour is related to the oscillations of the system. In case of the longer rod, the maximum normal force is obtained during compression and is equal to 2867 N, whereas, for the shorter rod, the maximum compressive force is equal to 4433 N. The forces with positive values are similar to compressive ones; hence, they were not included in the further work due to the small stress value and the fact that, in the case of tensile, they do not lead to buckling.

### 4.2. Composite Suspension System Rods

In the following steps, we performed numerical calculations of the composite rods separately, including modal analysis, static and dynamic buckling analyses, as well as static compression analysis and dynamic response analysis (to the loading according to suspension system impulse calculated in the aforementioned analyses)—for both rods with initial imperfection.

The analyses were performed firstly for the longer rod, for 8 stacking sequences presented in Section 3. The results showed that, for both static and dynamic loadings, the most beneficial results were obtained for four rods with axial plies placed on the outer side (stacking sequences (45/−45/902/02), (902/45/−45/02), (90/45/90/−45/02), and (45/90/−45/90/02)). They presented higher stability, strength, and stiffness compared with the other considered configurations. However, the differences between the four aforementioned stacking sequences were negligible, which suggests a minor influence of the placement and order of the 45°/−45° and 90° plies on the strength and stiffness of the construction.

Significant changes in the laminate ply orientations may cause delamination due to the creation of high shear stresses between the plies. Therefore, to eliminate the risk of delamination for the analyses of rod 2, we assumed a stacking sequence with the minor differences in consecutive ply orientations—(90/45/90/−45/02), where the maximum difference is 45°. In the other stacking sequences from the group of four best solutions, the differences reached 90°.

The order and methods of the calculation were the same for both rods.

**Figure 12:**
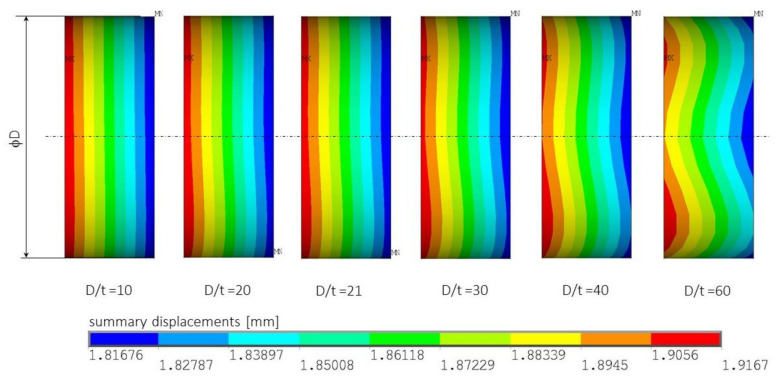
Distribution of summary displacement in a middle part of the laminate rod.

In the beginning, it should be pointed out that, considering the layer arrangement along with the rod thickness, they are not symmetrical and can cause non-obvious behaviour due to non-zero elements of the ABD laminate stiffness matrix responsible for the coupling between loads and deformations. Nevertheless, the considered structure is treated as a rod (not as a thin-walled shell structure), and, according to cross-section axes of symmetry, the layer arrangements are symmetrical. To prove the above, a simple numerical test was performed. Assuming the constant relation of L/D = 15.5 and different D/t = 10, …, 60 ratios (in the considered rod D/t = 21) for the rod with circular cross-section and layer arrangements No. 5 (c.f. Table 3), the compression test was performed, and summary displacements were determined. The results presented in Figure 12 show summary displacement on the side of the rods only in their middle part of length equal to c.a. 0.5 D. Comparing the displacement distribution, it can be easily noticed that, for D/t > 30, the displacement distributions are not constant as in the case of uniform compression—the effects of non-zero elements B_ij_, D_16_, and D_26_ of ABD laminate stiffness matrix.

#### 4.2.1. Modal Analysis

To begin with, we performed a modal analysis of each considered composite rod to determine their natural frequencies. In the following work, it enabled the investigation of possible resonance phenomena due to impact time concurrence with the natural period, as well as dynamic stability loss analysis.

The values of natural frequencies are presented in Table 3. The natural frequency for steel rod is significantly lower than for composite rods. The highest frequencies, and hence the lowest natural periods, were obtained for the last four stacking sequences.

#### 4.2.2. Static Buckling and Compression with Initial Imperfection

The composite rods research included static buckling and non-linear static compression analysis. There was an assumed initial geometrical imperfection for the calculations with a shape corresponding to buckling mode and amplitude equal to 5% of outer diameter. The composite strength was calculated by determining the failure index employing the Tsai–Wu failure criterion.

**Figure 13:**
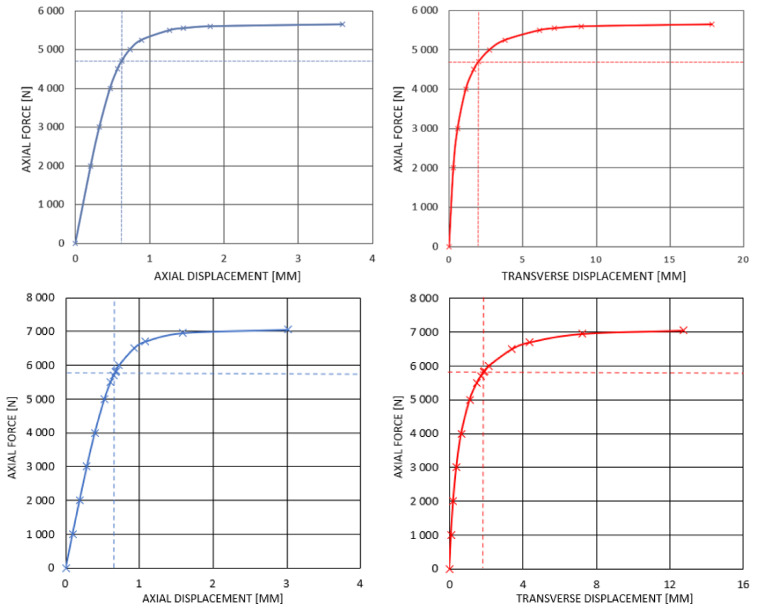
Axial and transverse displacement under static loading for rod 1 (stacking sequence 4 (0°/0°/45°/−45°/90°/90°)) and rod 2 (stacking sequence (90/45/90/−45/02)), respectively.

According to the numerical simulations, a loss of stability due to buckling will not occur. Before that, the initiation of the composite failure process will take place (Table 4 and Table 5). The force corresponding to the Tsai–Wu index equal to 1 is lower than the critical static force determined in buckling analyses. Furthermore, it is also lower than force corresponding to buckling of the rod with 5% imperfection (according to the definition, the loss of stability occurs when, for small loading increment, a substantial increment of displacement takes place). Figure 13 presents the axial and transverse displacement plots and compressive force for both considered constructions—stacking sequence 4 (0_2_/45/−45/90_2_) for rod 1 and stacking sequence 7 (90/45/90/−45/0_2_) for rod 2. Dashed lines indicate forces for which failure index reached 1.

**Figure 14:**
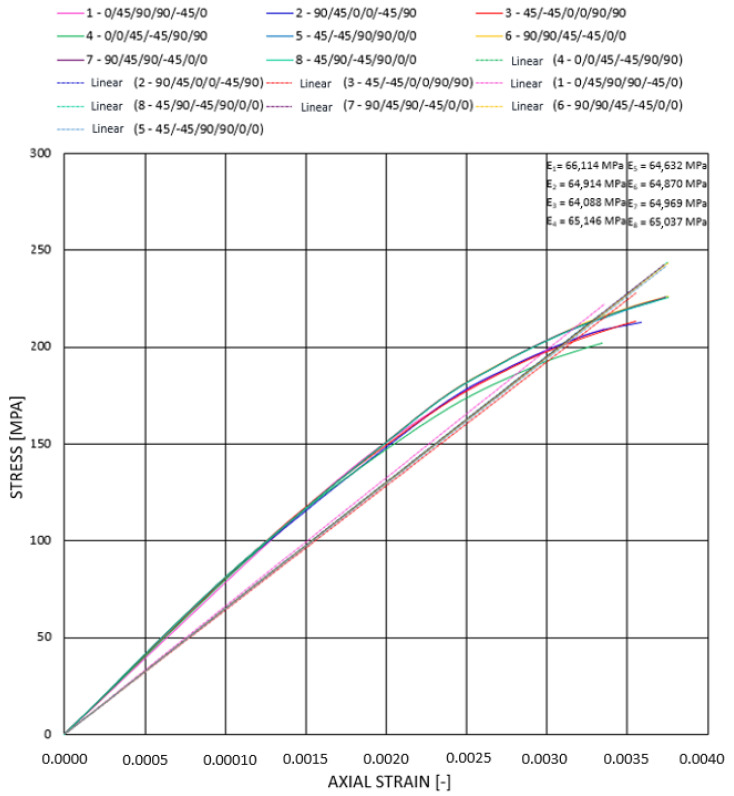
Stress–strain relationship for 8 stacking sequences (rod 1) under static compression.

Analogically, we performed analyses for each of the 8 stacking sequences for the longer rod. Figure 14 presents the stress–strain relationship for compression for all considered stacking sequences.

The character of the plot for all of the rods is similar and is a result of the complex nature of composite materials and inclusion of large displacement in the numerical calculations, and hence, stiffness recalculation in each iteration. Initially, to approximately 150 MPa, the plots for all rods are coincident. The differences occur above that value, and it is visible that the rods are divided into three groups. Then, the highest modulus of elasticity, hence, the highest stiffness, is observed for rods 1–3, whereas the lowest is for rod 4. It indicates the influence of stacking sequence on the stiffness of the construction. That is, since composite materials constitute a combination of materials of different properties, and longitudinal elasticity is a function of the elasticity modulus of each material separately. According to the theorem of mixtures, elasticity modulus for composite materials is dependent on the volume fraction of each material. Thus, it indicates the impact of stacking sequence on the resultant Young’s modulus. External layers, due to higher diameter, also pose higher volume in the rod compared with internal layers. Therefore, the largest influence on Young’s modulus and hence stiffness has the most external layers.

According to the Epoxycarbon UD396MPa characteristics and the results of performed numerical analyses of the rods with stacking sequence (0_6_), (45/–45)_3_, and (90_6_), the highest Young’s modulus is observed for composite with longitudinal layers. Its stiffness is 10 times higher than the stiffness of those with plies 45°/−45° and more than 20 times higher than that of those with plies 90°. Hence, the highest modulus is obtained for rods with longitudinal (0°) layers from the outside. Due to such significant differences in elasticity modules (of each layer separately), the placement of layers 45°/−45° and 90° does not significantly influence the resultant elasticity modulus.

The stress–strain plots were linearly approximated to obtain estimative Young’s modulus. These values oscillate between 64 and 66 GPa; hence, they are similar for all considered rods, which, compared with steels Young’s modulus at level 200 GPa, is a relatively small value and results in a significantly lower displacement for steel rod. However, taking into consideration specific Young’s modulus (Young’s modulus divided by the density) for composite rods, it is between 42.22 and 43.55 $\frac{\mathrm{GPa}}{\mathrm{kg}/m^{3}}$, whereas for steel, it is 25.45 $\frac{\mathrm{GPa}}{\mathrm{kg}/m^{3}},$ which constitutes a positive feature of carbon composite compared with classical construction material such as steel (Young’s modulus referenced to density is over 71% higher for Epoxycarbon UD365GPa).

**Figure 15:**
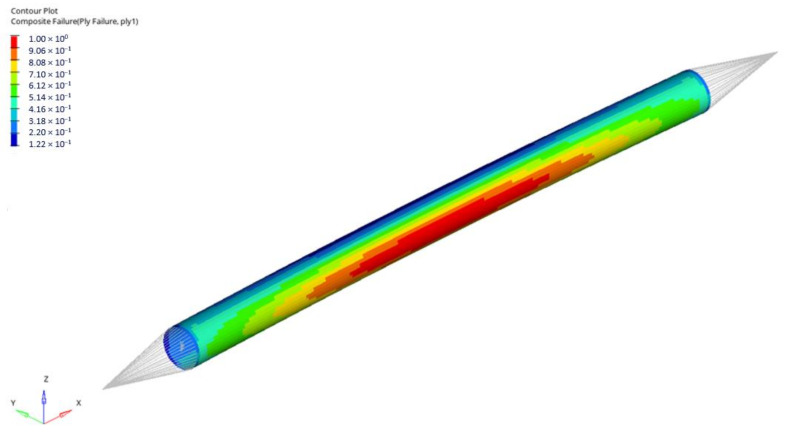
Tsai–Wu failure index map for rod 1 (stacking sequence no 1—Force 4 968 N)—1st layer failure.

In performed numerical analyses, the resultant forces were obtained corresponding to composite failure (according to Tsai–Wu failure criterion). The FI map for the first stacking sequence (0/45/90_2_/−45/0) for rod 1, corresponding to the failure index equal to 1, is presented below (Figure 15). The distribution of the obtained FI values is very similar for all considered stacking sequences. The most significant change is the number of plies in which the failure occurs; however, it is always a ply with axial fibre orientation because it carries the highest load.

The obtained critical static forces with other results for all stacking sequences for rod 1 are presented in Table 4.

The composite failure force was calculated assuming the first ply failure hypothesis. According to the analyses, in all considered cases, it was below critical static force value calculated with linear buckling analysis (for ideal straight rod), as well as the one determined for a rod with the initial geometrical imperfection of 5% (exemplary result for stacking sequence 4 is presented in Figure 12). It was observed that the highest composite failure forces are obtained for rod stacking sequences 5–8. Their common feature is the equivalent placement of axial laminate layers (0°)—in the outer part of the rod, whereas the only difference is the placement of the remaining four layers. The highest composite failure force equal to 5368 N was obtained for rod stacking sequence (90_2_/45/−45/0_2_) (12% higher than for stacking sequence 4 and 20% higher than for steel rod—in the case of steel, this value corresponds to yield strength). It is because fibre-reinforced composites are featured by the highest strength (for compression and tension) in the axial orientation (0°). The worst mechanical properties are obtained for transverse orientation (90°). The difference in the properties is very high—for the chosen composite compressive strength in the axial direction, it is equal to 893 MPa, whereas, in the transverse direction, it is equal to only 139 MPa. The difference is even more significant for tension—1979 MPa and 26 MPa, respectively. The difference in strength, depending on the layer orientation, explains the influence of stacking sequence on the general strength of the rod. The layer’s placement determines its cross-section and hence its area, which is largest for the outer layers. The highest value was obtained for stacking sequence (90_2_/45/−45/0_2_) and reached 5368 N, yet the differences within the rods with outer axial layers (stacking sequences 5–8) are negligibly small (not exceeding 0.3%). Shifting the axial layers to the middle results in a decrease in failure force by 6%, whereas separation of two axial plies to the farthest inner and outer results in a reduction by 7%. This indicates that the strength depends not only on their cross-section area, but also on whether they are directly adjoining. The highest differences in composite failure force are also relatively small and were obtained for the rod (0_2_/45/−45/90_2_), reaching 10%. This indicates that even the weakest composite rod is characterised by higher static carrying capacity than the steel one. All of the considered composite rods (stacking sequences) are capable of withstanding applied static load (due to a suspension system reaction force equal to 423 N for rod 1), and the ratio between composite failure force and actual load is very high and is equal to 11.4–12.7 (10.6 for steel rod). Yet, the largest difference between composite and steel rods is observed when comparing specific failure forces (failure force per material density). In case of composite rods, it reaches 3.54 $\frac{N}{\mathrm{kg}/m^{3}}$, whereas, for the steel rod, it is only 0.57 $\frac{N}{\mathrm{kg}/m^{3}}$, which means that, for the composite, that value is over 6 times higher.

Similarly, the obtained results for rod 2 are presented in Table 5.

In this case, the difference in the failure force is even higher than for the rod 1. The steel rod carries a 25% smaller load than the composite rod (for rod 1, the difference was 16%). The difference is even more prominent when considering failure force per material density—then, the failure force is 85% smaller for steel, which is very similar to the result obtained for rod 1 (84%).

Similarly, as previously, higher stiffness for compression was obtained for steel rod. This is due to the application of two axially oriented laminate layers—increasing this number would significantly improve the composite rod stiffness. However, again, recalculation of this value per density proves the benefit of applying CFRP laminate instead of steel alloy.

In all of the considered cases (for rod 1 and 2), the action of axial compressive force causes failure of one of the axially oriented layers (0°) (usually, the outer one first) under the action of compressive stresses exceeding the strength of the material. This is caused by the fact that the highest stiffness characterises axial layers, and hence, they carry the largest fraction of loading.

#### 4.2.3. Dynamic Loading with Triangular Impulse

**Figure 16:**
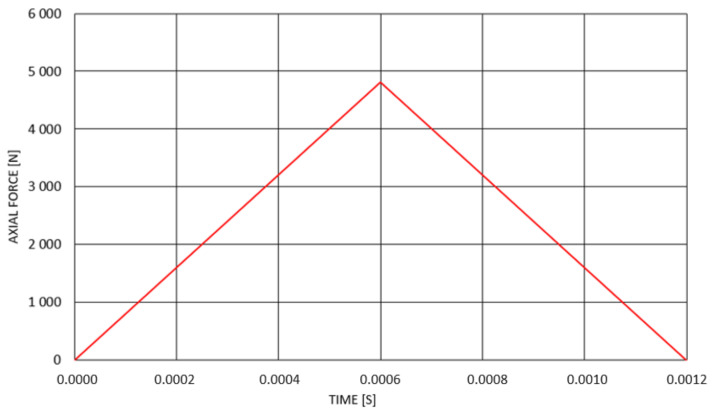
Triangular dynamic loading for composite rod 1 (0_2_/45/−45/90_2_).

The investigation of the behaviour of composite rods under the action of time-dependent triangularly shaped dynamic loading (Figure 16) was performed. The loading force was applied in the time corresponding to the natural period of each rod (obtained in performed modal analyses).

**Figure 17:**
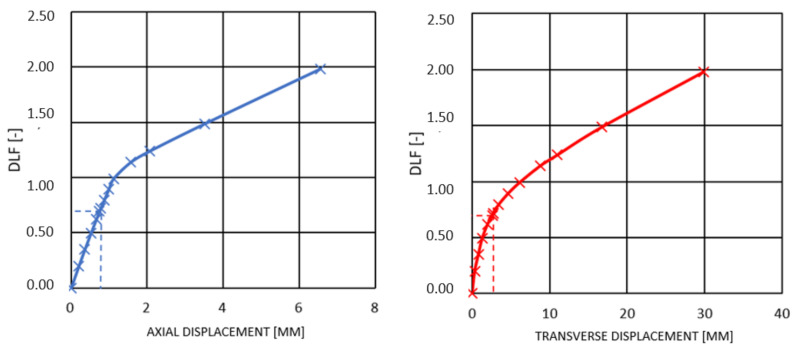
DLF as a function of longitudinal and transverse displacement for rod 1 (90/45/90/−45/0_2_).

In case of all considered rods and their stacking sequences, it was observed that, as previously, the composite failure process is initiated (Tsai–Wu index reaches 1) before the dynamic buckling phenomena are observed. According to Budiansky–Hutchinson’s criterion [11], a loss of dynamic stability occurs when for a small increment of loading, there takes place a rapid increment of deflection. Figure 17 presents an exemplary DLF (dynamic load factor) plot as a function of displacement in longitudinal and transverse direction for one of the rod 1 stacking sequences. The dashed lines indicate critical DLF corresponding to failure index equal 1 (approximately 0.7).

**Figure 18:**
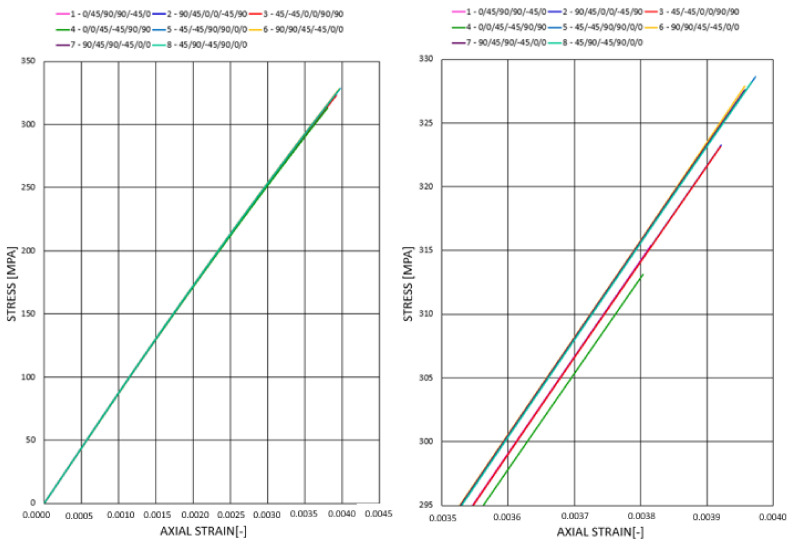
Stress–strain plot for dynamic compression.

Figure 18 presents the stress–strain plots for all stacking sequences under the influence of triangular dynamic impulse to the value of composite failure force. It is visible that the character and plot angle of inclination (and hence, longitudinal modulus of elasticity and stiffness) for all rods is more coincident than in static analysis. However, after rescaling the plots in the region close to the failure force, there was a noticeable division into 3 separate rods groups—similarly, as in the previous analysis, the highest longitudinal modulus of elasticity was obtained for rods 5–8, whereas the lowest was for rod stacking sequence 4. The values, however, are very similar for all cases, and the differences are negligibly small (the difference in the relative axial displacement for the best and the worst rod is not exceeding 0.5·10^−4^, which corresponds to 0.001 mm).

It can be observed that the plots are more linear than in the case of static compression. That is because applied dynamic loading is characterised by a very short impact time—the force increases rapidly and then rapidly decreases. During loading with external force inside the material, internal forces of interactions between material particles arise. However, in case of very rapid impulses, the particles do not keep up with reacting to the applied loading (impulse peak) since the changes in the internal structure occur slower. It follows that before the rods begin to “sense” the maximum impulse force, it begins to drop. Hence, the rods, in a way, “sense” the smaller load that is actually applied, and both their stiffness (elasticity modulus) and strength are enhanced compared with static loading.

**Figure 19:**
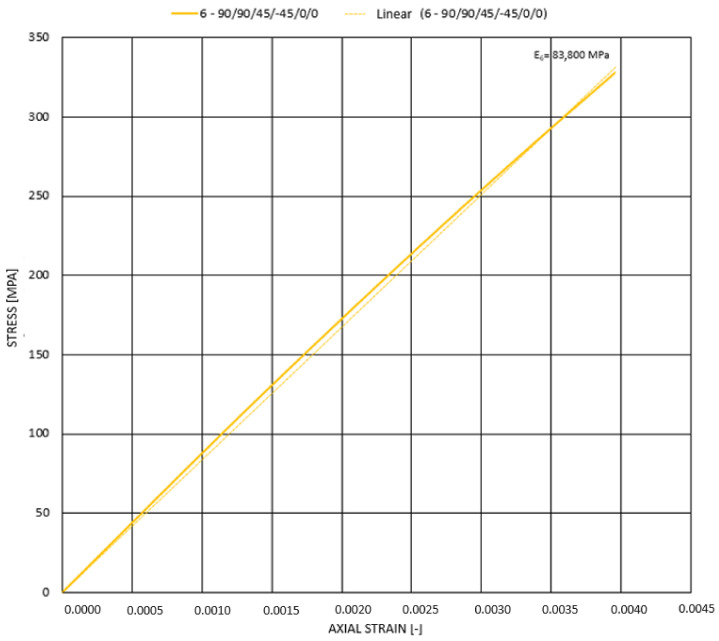
Stress-stain plot for dynamic compression of rod 1 (90_2_/45/−45/0_2_).

In this case, the linearly approximated longitudinal elasticity modulus reaches 83–84 GPa (Figure 19), which is an increase of over 26% compared with the characteristic of rods subjected to static loading. This means that the difference in specific elasticity modulus for composite and steel constructions is even more significant since these values are equal to 55 $\frac{\mathrm{GPa}}{\mathrm{kg}/m^{3}}$ (for composite) and 25 $\frac{\mathrm{GPa}}{\mathrm{kg}/m^{3}}$ (for steel).

**Figure 20:**
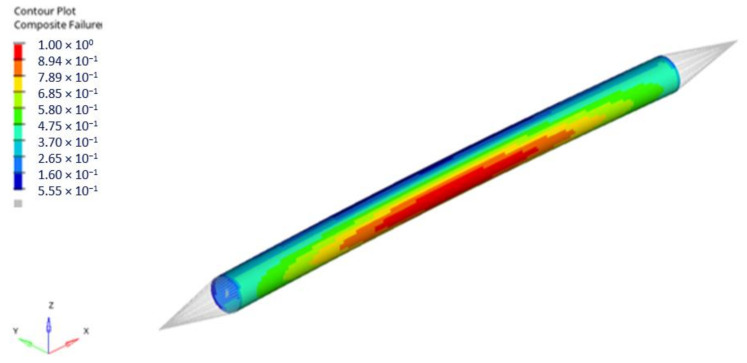
Tsai–Wu failure index maps for rod 1 (rod stacking sequence 1).

Figure 20 presents Tsai–Wu failure index maps for rod 1 for the first stacking sequence under dynamic loading.

Table 6 presents the results of the analyses for all considered stacking sequences of rod 1. The highest amplitude of dynamic pulse corresponding to DLFcr is obtained for rods 5–8. This time, the highest pulse amplitude value was reached for rod 6 and was equal to 12,440 N, which corresponds to the dynamic load factor according to Budiansky–Hutchinson’s criterion DLFcr at level 1.15. The same (the largest) DLFcr coefficient values were obtained for other rods from group 5–8. However, similarly, as in static analysis, composite material failure is initiated before the critical dynamic load is reached. The highest value of failure load was obtained for rod stacking sequence 5 and is equal to 7802 N. Yet, the differences between the failure loads for these rods are very low: below 1%.

Lower than for composite rods from group 5–8. The highest difference for composite and steel rods is observed for specific dynamic failure amplitude (failure amplitude per material density), where an amplification of over 8–fold occurs. Thus, this difference is even higher than in the case of static loading—specific dynamic failure amplitude for composite rods reaches 5.14 $\frac{N}{\mathrm{kg}/m^{3}}$, whereas for steel, it is only 0.60 $\frac{N}{\mathrm{kg}/m^{3}}$.

Table 7 presents the result for rod 2—for both composite (stacking sequence (90/45/90/−45/0_2_)) and steel rods, the dynamic critical amplitude is higher than the static critical force, which results in DLFcr higher than 1. In the case of the composite rod (stacking sequence (90/45/90/−45/0_2_)) the DLFcr is equal to 1.15, corresponding to the dynamic critical amplitude 14,632 N. Similarly, dynamic failure amplitude (according to the first ply failure and yield strength) is higher than static failure force—for the composite rod, the obtained value is equal 8064 N, whereas for steel, it is almost 50% less—4690 N. The difference is even more significant taking into consideration specific dynamic failure amplitude (recalculated per density), which, in the case of steel, is almost 89% lower (in the case of rod 1, the value was 88%).

Similarly, as in static analysis, the process of composite failure in all cases begins with plies of axial orientation due to its largest stiffness.

#### 4.2.4. Dynamic Loading with Suspension System Impulse

**Figure 21:**
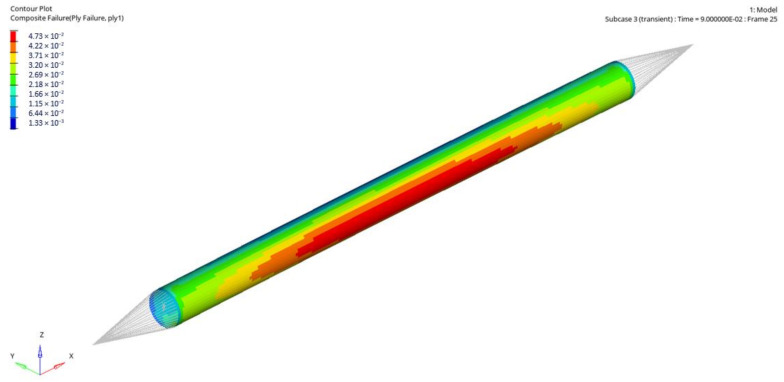
Tsai–Wu failure index map for suspension system dynamic pulse (rod 1, stacking sequence 1).

The last considered loading type was a force impulse according to the suspension system reactions due to passing a road bump, as in the previously presented impulse plots. The results of the analyses for rod 1 were gathered in Table 8. According to the finite element analysis, the rod with all the considered stacking sequences is capable of carrying the applied dynamic load (compressive-tensile impulse). For all of them, the Tai–Wu failure index was below 1. Figure 21 presents the Tsai–Wu failure index map for stacking sequence 1 of rod 1.

According to the analysis, the loading can also be carried by the steel rod in which maximum von Mises stresses reached 157 MPa, which is below material yield strength.

Under the action of dynamic loading, among all stacking sequences, the lowest axial and normal displacement was obtained for stacking sequences 5–8, whereas the lowest value was reached for rod 1 with 6th layup (90_2_/45/−45/0_2_). In case of axial displacement, the value was equivalent to the one obtained for layups 5 and 7 and was equal to 0.323 mm, whereas normal displacement for rod 6 was the lowest among all and reached 0.585 mm. Yet, the differences between stacking sequences from that group were very low (decimal fractions of a millimeter).

The performed analysis also showed that the rod with layer arrangement 6 (see Table 8) would carry the highest load of analogical character since the failure force and actual force ratio, in this case, is equal to 1.791, which corresponds to 5140 N in peak. This shows that the dynamic loading due to suspension system action is more demanding for the construction than the previously assumed triangular-impulse loading, where forces in peak reached 7783 N, which constitutes 51% more.

The results for rod 2 are presented in Table 9.

In case of the composite rod (stacking sequence (90/45/90/−45/0_2_)), there was no failure—the obtained Tsai–Wu failure index was smaller than 1. However, in case of steel, the applied loading resulted in exceeding the yield strength of the material—the maximum stress value reached 236 MPa; hence, it would not be able to carry the load safely. On the other hand, the composite rod would carry a 1.223 times higher loading, which would then result in the failure of the first axial ply of the laminate.

## 5. Conclusions

At present, carbon fiber reinforced composites has become increasingly popular in the automotive industry, and consequently, its application is being investigated, and the results are published. Yet, the number of articles concerning the application of composite materials in vehicles’ suspension system components and their optimisation is still limited.

During the research, we analysed the current (steel rod in suspension system) solution of the solar vehicle suspension system and the possibility of its improvement with the implementation of CFRP composite materials in place of upper control arm rods. We considered both static and dynamic loading types. The analysis showed that, in case of such rods, subjected primarily to axial loadings, the most suitable composite material would be uniaxial CFRP, due to its excellent mechanical properties in the axial direction—considering both strength for compression and tension, as well as Young’s modulus in fiber direction. The study provides an insight into the problem of suspension system components’ mass optimisation. In combination with an epoxy resin, such a composite may result in material strength exceeding 2000 MPa in the case of axial tension and 1000 MPa considering axial compression, simultaneously maintaining a high modulus of elasticity (at a level of 134 GPa) and allowing significant mass reduction (reaching, in this case, over 80%).

The analysis presented in this paper was mainly focused on steel rods compared with composite ones, checking the influence of layer arrangement as well. Our performed analyses showed that:

- The rods of such geometry are primarily subjected to composite failure rather than static or dynamic stability loss due to buckling (Tsai–Wu failure index reaches 1 before the occurrence of rapid deformations due to buckling)—the same relation was observed in case of steel rods.
- All of the considered rods can carry the considered static and dynamic loads.
- We observed an influence of the ply order on the stiffness and strength of the construction. The placement of axial layers (0°) is crucial. On the other hand, the arrangement of plies 45°/−45° and 90° does not have a significant impact.
- The highest results of failure forces, buckling loads, and estimative modulus of elasticity were obtained for stacking sequences with the outer placement of axial (0°) layers (farthest from the neutral axis in the case of bending deflection due to buckling)—configurations of layer denoted as 5–8.
- The longitudinal estimative modulus of elasticity referenced to the density of material proves its excellent stiffness capabilities even compared with steel (71% higher value obtained for the composite rod with reference to the steel).
- The failure force referenced to the material density is, respectively, in the case of static and dynamic loadings, over 6 and 8 times higher in favor of composite material (compared with the results for steel).
- We observed a noticeable (reaching even 45%) amplification in failure force value for dynamic loading with respect to static loading.
- In case of reaching the force corresponding to failure in all considered cases, always one of the axials (0°) plies fails first.
- Considering both (longer and shorter) composite rods with stacking sequence (90/45/90/−45/0_2_) under the action of static loading or dynamic pulse, the most crucial rod, taking into account construction strength, is the shorter one. In case of static compression, the failure load to actual load ratio equals 10.9 compared with 12.7 for rod 1, whereas, in the case of dynamic loading, the ratios are 1.6 and 1.8, respectively.

For future studies, an analysis of the influence of other loading types on the action of the suspension system components and the conduction of experimental testing has been planned.

## Figures and Tables

**Table 1:** Epoxycarbon UD395GPa material properties.

Young’s modulus in X direction	2.09∙10^5^	MPa
Young’s modulus in Y direction	9450	MPa
Poisson ratio	0.27	-
Kirchhoff’s modulus	5500	MPa
Tensile strength in direction X	1979	MPa
Tensile strength in direction Y	26	MPa
Compressive strength in direction X	−893	MPa
Compressive strength in direction Y	−139	MPa
Shear strength	100	MPa

**Table 2:** Rods’ stacking sequences (from inside out).

No	Stacking Sequence
1	(0/45/902/−45/0)
2	(90/45/02/−45/90)
3	(45/−45/02/902)
4	(02/45/−45/902)
5	(45/−45/902/02)
6	(902/45/−45/02)
7	(90/45/90/−45/02)
8	(45/90/−45/90/02)

**Table 3:** Modal analysis results.

No	Stacking Sequence	Longer Rod		Shorter Rod	
		Natural Frequency [Hz]	Natural Period [ms]	Natural Frequency [Hz]	Natural Period [ms]
1	(0/45/90_2_/−45/0)	822	1.22	939	1.06
2	(90/45/0_2_/−45/90)	822	1.22	937	1.07
3	(45/−45/0_2_/90_2_)	818	1.22	934	1.07
4	(0_2_/45/−45/90_2_)	803	1.25	916	1.09
5	(45/−45/90_2_/0_2_)	837	1.19	956	1.05
6	(90_2_/45/−45/0_2_)	840	1.19	959	1.04
7	(90/45/90/−45/0_2_)	839	1.19	958	1.04
8	(45/90/−45/90/0_2_)	838	1.19	957	1.04
9	steel	541	1.85	632	1.60

**Table 4:** Results for non-linear static compression of rod 1.

No	Stacking Sequence (from Inside out)	Critical Static Force According to LBA [N]	Critical Static Force According to NLA [N]	Composite Failure Force According to FPP [N]	First Ply Failure [–]	Failure Force to Actual Force Ratio [–]	Specific Failure Force [N/(kg/m^3^)]
1	(0/45/90_2_/−45/0)	10,350	5700	4968	1 (0°)	11.7	3.27
2	(90/45/0_2_/−45/90)	10,342	5800	5109	4 (0°)	12.1	3.37
3	(45/−45/0_2_/90_2_)	10,265	5700	5063	3 (0°)	12.0	3.34
4	(0_2_/45/−45/90_2_)	9874	5600	4811	1 (0°)	11.4	3.17
5	(45/−45/90_2_/0_2_)	10,752	6000	5362	5 (0°)	12.7	3.53
6	(90_2_/45/−45/0_2_)	10,817	6000	5368	5 (0°)	12.7	3.54
7	(90/45/90/−45/0_2_)	10,805	5950	5359	5 (0°)	12.7	3.53
8	(45/90/−45/90/0_2_)	10,771	5950	5354	5 (0°)	12.7	3.53
9	steel	5213	4550	4472	n/a	10.6	0.57

LBA—linear buckling analysis—eigenvalue problem. NLA—non-linear analysis—large displacement with initial geometrical imperfection. FPP—first ply failure hypothesis for laminates (Tsai–Wu criterion).

**Table 5:** Results for non-linear static compression of rod 2.

No	Stacking Sequence (from Inside out)	Critical Static Force According to LBA [N]	Critical Static Force According to NLA [N]	Composite Failure Force According to FPP [N]	First Ply Failure [–]	Failure Force to Actual Force Ratio [–]	Specific Failure Force [N/(kg/m^3^)]
1	(90/45/90/−45/0_2_)	12723	6700	5839	6 (0°)	10.9	3.85
2	steel	5243	4650	4355	n/a	8.12	0.56

**Table 6:** Results of dynamic loading analysis with the triangular impulse for rod 1.

No	Stacking Sequence	DLFcr [–]	Amplitude of Dynamic Pulse Corresponding to DLFcr [N]	Amplitude of Dynamic Pulse Corresponding to FFP [N]	First Ply Failure [–]	Specific Dynamic Failure Amplitude [N/(kg/m^3^)]
1	(0/45/90_2_/−45/0)	1.05	10868	7 487	1 (0°)	4.93
2	(90/45/0_2_/−45/90)	1.10	11376	7 674	4 (0°)	5.05
3	(45/−45/0_2_/90_2_)	1.10	11292	7 670	3 (0°)	5.05
4	(0_2_/45/−45/90_2_)	1.05	10368	7 433	1 (0°)	4.89
5	(45/−45/90_2_/0_2_)	1.15	12365	7 802	5 (0°)	5.14
6	(90_2_/45/−45/0_2_)	1.15	12440	7 783	5 (0°)	5.13
7	(90/45/90/−45/0_2_)	1.15	12426	7 776	5 (0°)	5.12
8	(45/90/−45/90/0_2_)	1.15	12387	7 790	5 (0°)	5.13
9	steel	1.1	5734	4 696	n/a	0.60

**Table 7:** Results of dynamic loading analysis with the triangular impulse for rod 2.

No	Stacking Sequence	DLFcr [–]	Amplitude of Dynamic Pulse Corresponding to DLFcr [N]	Amplitude of Dynamic Pulse Corresponding to FFP [N]	First Ply Failure [–]	Specific Dynamic Failure Amplitude [N/(kg/m^3^)]
1	(90/45/90/−45/0_2_)	1.15	14 632	8 064	6 (0°)	5.31
2	steel	1.10	4 791	4 690	n/a	0.60

**Table 8:** The results of dynamic analysis due to suspension system impulse—rod 1.

No	Stacking Sequence	Tsai–Wu Failure Index [–]	Maximum Axial Displacement [mm]	Maximum Normal Displacement [mm]	Failure Load Amplitude to Actual Load Ratio [–]	First Ply Failure
1	(0/45/90_2_/−45/0)	0.473	0.329	0.639	1.647	1 (0°)
2	(90/45/0_2_/−45/90)	0.477	0.329	0.640	1.705	4 (0°)
3	(45/−45/0_2_/90_2_)	0.480	0.329	0.647	1.691	3 (0°)
4	(0_2_/45/−45/90_2_)	0.485	0.333	0.696	1.603	1 (0°)
5	(45/−45/902/0_2_)	0.468	0.323	0.593	1.789	5 (0°)
6	(90_2_/45/−45/0_2_)	0.464	0.323	0.585	1.791	5 (0°)
7	(90/45/90/−45/0_2_)	0.465	0.323	0.588	1.788	5 (0°)
8	(45/90/−45/90/0_2_)	0.467	0.324	0.593	1.787	5 (0°)
9	steel	n/a	0.114	0.187	1.51	n/a

**Table 9:** The results of dynamic analysis due to suspension system impulse—rod 2.

No	Stacking Sequence	Tsai–Wu Failure Index [–]	Maximum Axial Displacement [mm]	Maximum Normal Displacement [mm]	Failure Load to Actual Load Ratio [–]	First Ply Failure
1	(90/45/90/−45/0_2_)	0.810	0.329	0.639	1.647	1 (0°)
2	steel	n/a	0.329	0.640	1.705	4 (0°)

## Data Availability

The data presented in this study are available on request from the corresponding author.
